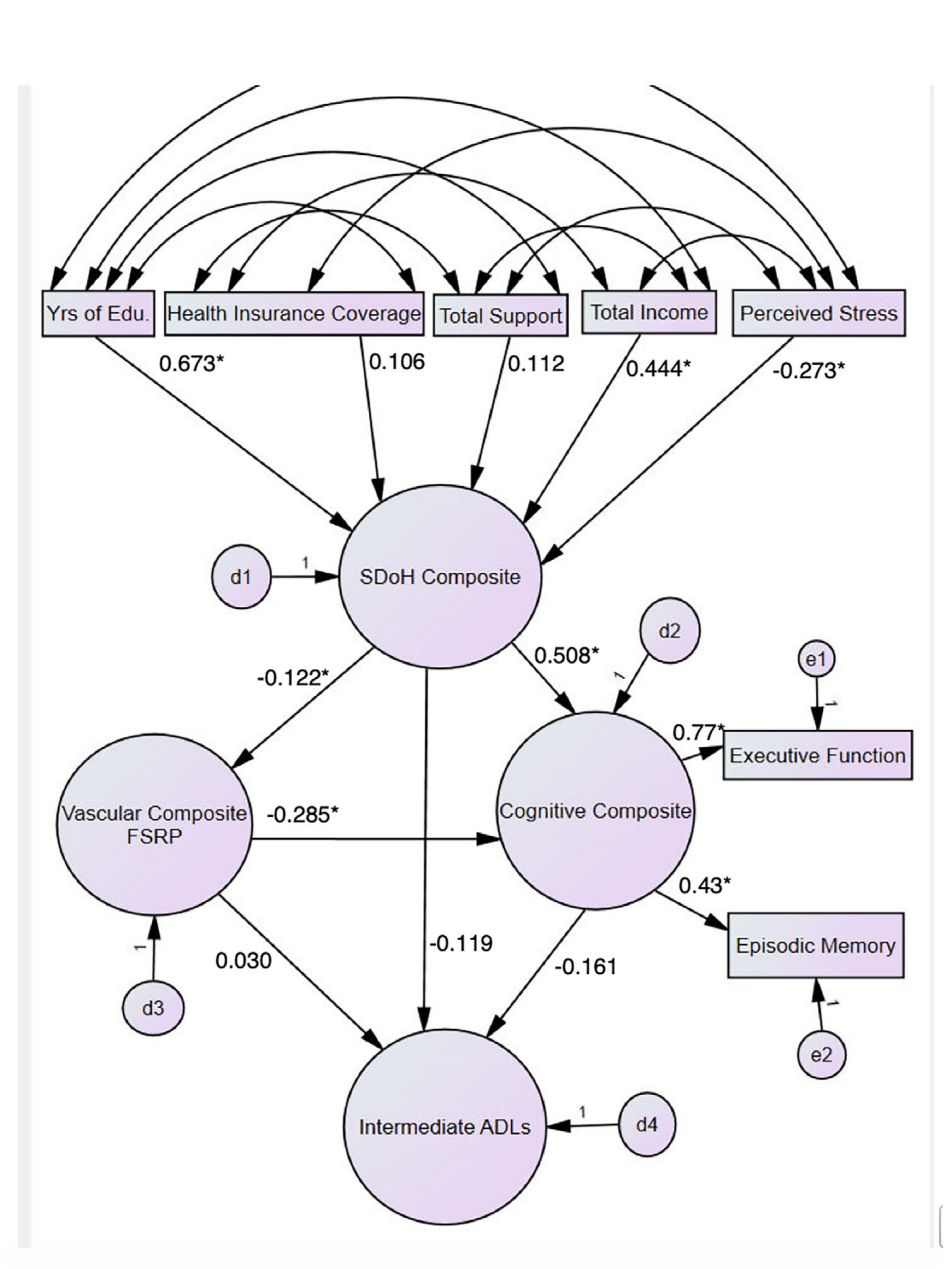# Interrelationships of social determinants with cognition, vascular health, and physical functioning

**DOI:** 10.1002/alz.088011

**Published:** 2025-01-03

**Authors:** Priscilla A Amofa‐Ho

**Affiliations:** ^1^ University of Florida, Department of Clinical and Health Psychology, Gainesville, FL USA

## Abstract

**Background:**

The primary aim of the current analysis was to evaluate the cumulative effect of different social determinants of health (SDoH) factors on vascular burden, cognition, and physical functioning.

**Method:**

We conducted a secondary data analysis of the MIDUS 2 cross‐sectional study. Participants were ages 55 and above. Measures derived from the Framingham Stroke Risk Profile, Brief Test of Adult Cognition by Telephone, and MOS‐36 were used to represent vascular burden, cognition, and intermediate activities of daily living, respectively. SDoH variables included education, income, health insurance, stress, and support from family and friends. Associations were evaluated using composite‐based structural equation model (c‐SEM) embedded in an overall.

**Result:**

Among cognitively normal adults (N = 568; mean age = 64.6), higher education, less stress, and higher income significantly contributed to the SDoH composite. Better standing in SDoH was associated with better physical functioning (beta = ‐0.21, p<0.001) and higher cognition (beta = 0.54, p<0.001). Significant direct effects of SDoH on vascular burden (beta = ‐0.12, p<0.05) and cognition (beta = 0.51, p<0.001), and of vascular burden on cognition (beta = ‐0.28, p<0.001) were found. Mediation analysis indicated that the unique effects of SDoH on cognition remained significant after controlling for vascular burden (beta = 0.04, p<0.05). After accounting for vascular burden and cognition, SDoH did not have a significant unique effect on physical functioning.

**Conclusion:**

Our results support the disablement process that suggests that factors outside of the disease model (like SDoH factors) impact both the underlying diseases that lead to physical and functional limitation, all of which are associated with dementia/cognitive decline.